# A Sanctioned Encampment as a Strategy for Increasing Homeless Veterans’ Access to Housing and Healthcare During the COVID-19 Pandemic

**DOI:** 10.1007/s11606-023-08124-4

**Published:** 2023-06-20

**Authors:** Ippolytos Kalofonos, Matthew McCoy, Lisa Altman, Lillian Gelberg, Alison B. Hamilton, Sonya Gabrielian

**Affiliations:** 1 HSR&D Center for the Study of Healthcare Innovation, Implementation & Policy (CSHIIP), VA Greater Los Angeles Healthcare System, Los Angeles, CA USA; 2 UCLA/VA Center for Excellence for Veteran Resilience and Recovery in Homelessness and Behavioral Health, Los Angeles, CA USA; 3 Center for Social Medicine and Humanities, Jane and Terry Semel Institute for Neuroscience and Human Behavior, David Geffen School of Medicine, UCLA, Los Angeles, CA USA; 4 UCLA International Institute, Los Angeles, CA USA; 5 Department of Psychiatry and Biobehavioral Sciences, David Geffen School of Medicine, University of California, Los Angeles, CA USA; 6 Department of Medicine, David Geffen School of Medicine, University of California, Los Angeles, CA USA; 7 Department of Family Medicine, David Geffen School of Medicine, University of California, Los Angeles, CA USA

**Keywords:** encampment, homelessness, veteran, COVID-19, ethnography

## Abstract

**Background:**

The COVID-19 pandemic intersected with a housing crisis for unsheltered Veterans experiencing homelessness (VEHs); congregate settings became high risk for viral spread. The VA Greater Los Angeles responded by creating the Care, Treatment, and Rehabilitation Service (CTRS), an outdoor, low-barrier-to-entry transitional housing program on VA grounds. This novel emergency initiative offered a protected outdoor environment (“sanctioned encampment”) where VEHs lived in tents and had access to three meals a day, hygiene resources, and health and social services.

**Objective:**

To identify contextual factors that supported and impeded CTRS participants’ access to healthcare and housing services.

**Design:**

Multi-method, ethnographic data collection.

**Participants:**

VEHs residing at CTRS, CTRS staff.

**Approach:**

Over 150 hours of participant observation were conducted at CTRS and at eight town hall meetings; semi-structured interviews were conducted with 21 VEHs and 11 staff. Rapid turn-around qualitative analysis was used to synthesize data, engaging stakeholders in iterative participant validation. Content analysis techniques were used to identify key factors that impacted access to housing and health services among VEHs residing in CTRS.

**Key Results:**

Staff varied in their interpretation of CTRS’ mission. Some conceptualized access to health services as a central tenet, while others viewed CTRS as an emergency shelter only. Regardless, staff burnout was prevalent, which lead to low morale, high turnover, and worsened access to and quality of care. VEHs endorsed trusting, long-term relationships with CTRS staff as paramount for facilitating access to services. Though CTRS addressed basic priorities (food, shelter, etc.) that traditionally compete with access to healthcare, some VEHs needed on-site healthcare services, at their tents, to access care.

**Conclusions:**

CTRS provided VEHs access to basic needs and health and housing services. To improve access to healthcare services within encampments, our data suggest the value of longitudinal trusting relationships, adequate staff support, and on-site health services.

## INTRODUCTION

Veterans experiencing homelessness (VEHs) face poor access to health services.^1–3^ Compared to their housed peers, VEHs have higher rates of physical illness, mental illness, and substance abuse; they are more likely to utilize Emergency Departments (EDs), be admitted to hospitals at younger ages, and have longer, more costly stays.^4^ VEHs have higher age-adjusted mortality^5^ and chronic disease rates than the general population. These disparities worsened during the 2019 coronavirus (COVID-19) pandemic, which rendered congregate housing and shelter settings high-risk for viral spread.^6,7^ Veterans Health Administration (VHA) facilities around the nation developed novel shelter practices during the pandemic, but little is known about the impacts of these housing innovations on VEHs’ access to care.

Before the pandemic, several approaches were developed to improve access to care for individuals experiencing unsheltered homelessness, i.e., living in locations not intended for human habitation.^8^ Within VHA, Homeless Patient-Aligned Care Teams (HPACTs)—comprehensive integrated primary care tailored for VEHs—has improved care.^3,9–12^ While HPACTs are housed in brick-and-mortar facilities, *street medicine*, an approach delivering care to unsheltered people where they reside, has emerged as another service-delivery model.^13–15^ During the pandemic, the VA Greater Los Angeles Healthcare System (VAGLAHS) developed a similar model within an encampment-based environment. 

Encampments have proliferated in US communities due to affordable housing shortages,^16^ anti-homeless laws, and government tolerance for out-of-sight encampments.^17^ In response to risks posed to unsheltered VEHs by the pandemic, compounded by escalating numbers of encampments in its community, VAGLAHS established the Care, Treatment, and Rehabilitation Service (CTRS) in April 2020. CTRS is an outdoor, low-barrier-to-entry transitional housing initiative on West Los Angeles VA medical center grounds (WLAVA). It began as a protected outdoor environment (“sanctioned encampment”) where VEHs lived in tents and were linked to healthcare and housing services at the medical center.

Given the unprecedented nature of this encampment intervention and concomitant opportunity for iterative process improvement, multi-level partnerships were built among CTRS Veterans, staff, and leadership; researchers and clinicians from the University of California (UCLA)/VA Center of Excellence (COE) for Veteran Resilience and Recovery; and facility-level leadership. Anthropologists using ethnographic methods were embedded in the encampment to identify and present Veteran and staff concerns to decision-makers. Quality improvement (QI) methods were used to improve services.^18^ This article documents the challenges and successes of an improvised response to a public health emergency that aimed to provide access to healthcare and housing services to VEHs but lacked clarity in organizational goals around the role of delivering healthcare services.

Access to care is conceptualized as the potential ease interacting with a broad array of service providers and includes self-reported, subjective perceptions of access.^19^ Perceived access may be a strong predictor of utilization and is particularly important with unhoused individuals who often feel unwelcome in traditional service settings.^20,21^ This article describes contextual factors supporting and impeding VEHs’ access to services in CTRS, highlighting the role of access to healthcare, and uses these data to suggest areas for improvement as the pandemic evolves. These data may inform novel housing models addressing access to services for this vulnerable population beyond the pandemic. A supported encampment, while not an ideal living situation, can serve as a temporary housing option for some unsheltered individuals while they establish a sense of stability, cultivate relationships, and ultimately access healthcare and permanent housing.

## METHODS

### Setting

CTRS was enacted in April 2020, when escalating COVID-19 rates intersected with high homelessness rates in Los Angeles. At the pandemic’s outset, 66,436 individuals were unsheltered countywide,^22^ including 3700 Veterans.^23^ CTRS was on the WLAVA campus, ½ mile from buildings where emergency, outpatient, and inpatient clinical services are offered.

CTRS was open to healthcare-eligible VEHs needing shelter and their spouses/partners via self-referral or referral from VA programs. Entrance requirements included a negative COVID-19 symptom screen, agreement to COVID test within 24 h, and a behavioral contract (consistent with a harm reduction approach, on-site intoxication was tolerated in the absence of behavioral disturbance). Table 1 summarizes CTRS infrastructure, staffing, and programming. Beginning as tents in a parking lot, CTRS transitioned to 100 raised platforms in a grass field monitored by 24/7 security. Though CTRS was designed as a short-term shelter, VEHs could initially stay as long as they needed. Veteran town hall meetings facilitated VEH input into the program and its development.

**Table 1:** Core Components of CTRS

Infrastructure	Onsite staffing	Programming
• Tent on a raised pallet • Sleeping cot, sleeping bag/blankets, pillow • Clothing • Personal locker • 24/7 contracted security • 3 meals/day • Unlimited water • Showers • Portable toilets and handwashing stations • Transportation to laundry facility • Electronic tablet and cell phone for resident use • Weekly COVID-19 surveillance testing • Intermittent vaccination clinics	• 2 full-time social workers • 2 full-time peer supports • Biweekly primary care and triage • Part-time preventive medicine physician • Part-time occupational therapist	• Substance use groups • Healthy teaching kitchen • Storytelling group • Music therapy group • Acupuncture • Veteran town halls

While staffing varied over time, at least two social workers and two Veteran peer specialists were assigned to CTRS. A preventive medicine physician provided care management for the first 7 months. The initiative provided care coordination, COVID-19 surveillance, chaplain services, occupational therapy, and twice monthly on-site urgent care services from VA primary care providers.^24^ To access routine ambulatory care, Veterans were referred to brick-and-mortar clinical services or virtual visits in their tents via a CTRS-provided tablet or personal device. On-site ancillary services included a healthy teaching kitchen, acupuncture, storytelling groups, and substance abuse groups.

### Design

Ethnography is a study design and method emphasizing long-term immersion within a field site.^25^ Data were collected using participant observation and interviews.^26^ Participant observation entailed involvement at CTRS in daily routines, development of ongoing relations with residents and staff, and^27^ documentation in written fieldnotes.^25,28^

### Data Sources

Data collection was part of an initiative to iteratively improve care for unsheltered VEHs during the pandemic. Relevant Institutional Review Boards deemed all procedures QI. Veterans were recruited via purposive sampling with attention paid to diversity in race, ethnicity, age, and gender. All staff were invited for interviews. Informed consent was obtained to conduct and digitally record interviews and to review Veterans’ EHRs for demographic and diagnostic information. The demographics and diagnoses of all Veterans over the data collection period were characterized using administrative data (from VA’s Corporate Data Warehouse and homeless registry).

The co-first authors, using physical distancing and personal protective equipment, conducted 55 weekly on-site participant-observation visits (150+h, 400+pages of unstructured fieldnotes) and observed eight Veteran town hall meetings (60 min each) from 9/1/2020 to 10/1/2021. These data were integrated with semi-structured face-to-face interviews (60–90 min each) with 21 unique Veterans and follow-up interviews with 7 long-stay Veterans (<180 days). Semi-structured interviews were also conducted with 11 CTRS staff with 6 follow-up interviews (60 min each). Follow-up interviews captured perspectives on encampment changes.

### Data Collection

Observations and quotations were written down contemporaneously by both anthropologists in a joint fieldnote developed after each visit to achieve consensus in ethnographic observations.^28^ Fieldnotes from the first 2 months (8 visits) guided the drafting of semi-structured interview questions corresponding to key concerns of VEHs and staff. VEHs were interviewed regarding: experiences accessing VA health and housing services; satisfaction with CTRS’ facilities, care, and services; and recommendations for improvement. To contextualize these findings, age, gender, race, ethnicity, and presence/absence of common mental health and substance use diagnoses of interviewed participants were abstracted from the EHR. CTRS staff were interviewed regarding: perceived purpose of CTRS; experiences in CTRS; and recommendations for improving service access.

### Data Analysis

Recorded interviews were professionally transcribed. Fieldnotes and interview transcripts were summarized and integrated using rapid turn-around analysis.^29^ Key stakeholders (e.g., QI team, CTRS management/staff, and COE researchers/clinicians) were engaged in iterative, real-time participant validation of qualitative data interpretation during a weekly QI meeting. Through co-first author consensus, a codebook was created to organize and facilitate analysis. Codes were derived from categories in the interview guides and emerging from interview transcripts and fieldnotes using content analysis principles of condensing texts into meaningful categories.^30,31^ The co-first authors coded using ATLAS.ti and identified key factors impacting access to housing and health services. The larger QI team reviewed and validated salient findings.

## Results

### Sample Characteristics

From 4/1/2020 to 10/1/2021, 381 unique Veterans were admitted to CTRS, with 110 (28%) admitted more than once (Table 2). Most residents were unsheltered or in temporary residences prior to the pandemic. The majority (59%) carried serious mental illness diagnoses (SMI, i.e., psychotic disorder). Nearly 52% had co-occurring SMI and substance use disorders. Average length of stay was 35 days (range: 1 day to over 1 year). The co-first authors interfaced with all VA-employed CTRS staff during the study period. Most staff (86%; *n* = 11) agreed to interviews.

**Table 2:** Characteristics of CTRS Residents

Demographic	Interview sample	All CTRS residents
Age (mean, SD, in years)	49, 15	54, 12.8
Age range in years	32–79	24–80
Gender		
Male (*N*, %)	18, 86%	358, 94%
Female (*N*, %)	2, 10%	23, 6%
Transgender female-to-male	1, 5%	n/a
Ethnicity		
Hispanic/Latino (*N*, %)	2, 10%	53, 14%
Non-Hispanic/Latino (*N*, %)	19, 90%	319, 84%
Unknown	0	9, 2%
Race^*^		
White (*N*, %)	12, 57%	197, 52%
Black (*N*, %)	8, 38%	155, 41%
Asian (*N*, %)	1, 5%	6, 2%
Native Hawaiian/Pacific Islander (*N*, %)	1, 5%	2, 1%
Native American	0	6, 2%
Unknown	0	15, 2%
Mental illness diagnosis^#^		339, 89%
PTSD (*N*, %)	9, 43%	
Psychotic disorder (*N*, %)	8, 38%	
Mood disorder (*N*, %)	5, 24%	
Personality disorder (*N*, %)	2, 10%	
Anxiety	1, 5%	
Substance use diagnosis^#^		309, 81%
Methamphetamine (*N*, %)	11, 52%	
Marijuana (*N*, %)	10, 48%	
Alcohol (*N*, %)	9, 43%	
Cocaine (*N*, %)	4, 19%	
Opioid (*N*, %)	3, 14%	

^*^One Veteran reported 2 races

^#^Most Veterans had multiple diagnoses; specific diagnoses not available for “All CTRS Residents”

### Overview of Qualitative Findings

By providing a safe and stable, if temporary, home, CTRS addressed fundamental needs impeding access to health and housing. The cultivation of trusting relationships and provision of onsite health services were additionally identified as key facilitators to accessing services. However, there was lack of clarity about the overall purpose of CTRS: whether to focus on housing exclusively or to prioritize health services as well. Consequently, CTRS’ mission was variably interpreted by Veterans and staff. This ambiguity combined with chronic understaffing led staff to feel unsupported and contributed to experiences of burnout.

### CTRS Addressed Needs that Can Impede Access to Care

Veterans regularly stated CTRS was their first “home” in years. Having a home and food gave Veterans a stable base from where they could prioritize their care needs. One Veteran explained: “I’ve built up stronger support, stronger communication…that I didn’t have before because I didn’t have a central location…Having a home is to stay safe.” Another Veteran said CTRS allowed her to engage in therapeutic hobbies while also addressing health issues: “ I’ve got a home…I’ve been able to be more productive…I do some planting…[and] a lot of hospital stuff too.” Many Veterans reported this sense of a safe home contributed to their ability to access services.

Veterans appreciated that CTRS lacked many restrictions of other housing programs, enabling them to access services on their own time. An elderly Veteran said he could “exhale” without the time pressure common in transitional housing: “I’m getting more stabilized. I don’t have them breathing down my neck with deadlines…I don't have the…countdown, six months, three months…just stability. It’s refreshing.”

### Trusting Relationships Enabled Access to Services

The cultivation of trusting relationships was described by several CTRS staff and nearly all Veterans as the first step in establishing access to services. This included relationships between Veterans and VA staff and among Veterans. Staff knew many Veterans at CTRS had prior negative experiences with VA services and often felt VA had let them down. One staff member explained: “Develop relationships, have the care people need, whether it’s substance abuse, mental health, primary care. Help people navigate and connect to [ housing services]…OK, let’s repair your relationship with the VA…It’s the foundation of good healthcare.” The process of developing trusting relationships entailed, another staff member said: “[Giving Veterans] the time and the space and the support to figure out what it is they want.” To develop relationships increasing access to care, staff emphasized the importance of patience and consistent, longitudinal engagement.

Veterans also described that relationship development with providers facilitated access to healthcare. One Veteran expressed the desire to have trusted providers visit his tent: “Just to have somebody come out and sit down and, even if it’s just to listen to us bitch about whatever. The interpersonal connection…does a lot to lift people’s spirits and to motivate people to progress and move forward.”

Veterans emphasized the importance of developing relationships with each other as the foundation of a supportive home that then facilitated access to services in various ways. Two Veterans discussed a friendship they formed in CTRS after they “both got thrown out” of a program for sobriety violations. Another Veteran described CTRS as a “safe spot” for Veterans, asserting, “we [i.e. Veterans] are the support [for each other].” He articulated a shared camaraderie among unhoused Veterans: “I’d still put my life on the line with that man…He’s a Vet…He’s disturbed like we’re all disturbed…Would you not be disturbed if you were denied an existence?” This testimonial demonstrates the concern Veterans had for each other. This concern was often translated into active encouragement to seek services. Notably, when COVID vaccinations became available, several well-respected Veterans volunteered to publicly receive injections and many Veterans followed suit.

### Some Veterans Needed On-Site Services to Access Care

Existing on-site clinical services were limited to twice-monthly urgent care visits. While these were popular, Veterans were expected to seek their own care: telecare from the encampment when many services went virtual at the start of the pandemic or in-person when departments reopened. While some Veterans used brick-and-mortar VA services, Veterans and CTRS staff reported others, particularly those with complex needs, struggled to access them. Wait times, cancelations, distance (particularly for Veterans with disabilities), anxiety provoked by clinical settings, and technical difficulties with virtual visits (via loaner tablet or personal device) were obstacles to accessing care and developing relationships with providers. These non-COVID-19-related barriers were amplified by the pandemic. Consequently, Veterans increasingly requested on-site care at their tents. One shared: “Instead of making us fend for ourselves…I’m medically disabled. I can’t go walk to work. If I could, I’d still be in the military.” A Veteran worried about a neighbor with a mouse-infested tent who seemingly had given up asking for help: “They’re not bringing him healthcare, which is what he needs.” Another Veteran voiced a common concern for those with psychiatric symptoms: “Get better mental health services for them.” Mental health services were not offered onsite; this led to increased behavioral disturbances and left VA police as first-responders. Police presence upset many Veterans.

### CTRS’ Mission Was Variably Interpreted by Veterans and Staff

Due to the improvisational nature of an intervention developed during an emergent pandemic, there was a lack of clarity and consensus about CTRS’ purpose. While some staff viewed enhancing access to healthcare services as a central mission, others felt offering healthcare services and developing relationships were beyond the scope of providing emergency shelter. Concerns arose around inadequate staffing and that Veterans may lose motivation to work towards permanent housing if healthcare was prioritized. These staff conceptualized CTRS as a temporary shelter and focused on quickly transitioning VEH to alternative housing (e.g., motels). In the words of one administrator: “There should be a time limit because we have to keep this as a progression and transition towards something.” Another expressed: “none of these people will seek services on their own if they are just allowed to be here, getting high,” and “a lot of these guys aren’t going to go anywhere until they’re forced.” These staff emphasized housing plans and brief CTRS stays. Discharges for rule violations and/or disruptive behavior were described as incentives for Veterans to practice self-responsibility and to transition into better housing. A robust version of person-centered care was eclipsed by a narrower approach, emphasizing CTRS as an emergency housing setting, not a clinical program. In part, this orientation emerged in response to understaffing, staff turnover, and burnout.

### Staff Burnout Worsened Access to Care

Staffing at CTRS was difficult from the start. CTRS was launched early in the pandemic when most employees were teleworking. While some embraced the opportunity to serve vulnerable Veterans during a crisis, many were daunted by the prospect of working face-to-face with Veterans with complex challenges. An administrator explained the challenge: “When you're out in the elements, without walls, I think people are fearful…of our Veterans…I understand why…They can be unpredictable…our Veterans are under the influence, are extremely mentally ill, and that can be scary.”

After about 1 year, staff felt overwhelmed by maintaining the daily operations of the encampment while attending to Veterans’ needs, from basic necessities to complex healthcare disorders. Given the novelty of the outdoor encampment, staff and Veterans improvised responses to challenges, from weighing down tents with sandbags against strong winds to offering cooling fans during heat waves. The same staff linking Veterans to health and housing services also pitched new tents and cleaned used tents, which included discarding spoiled food, excrement, and broken glass.

Compounding the physically taxing nature of this work, frequent interpersonal conflicts on-site between VEH and between VEH and staff worsened workplace wellness. Staff bore the brunt of disagreements often escalating to personal insults/threats. Beyond fear of COVID-19 infection, some staff described distress about their work: “I’m anxious almost every day. [Am I] going to get that phone call today that a tent is on fire?…It’s also that depression of feeling like, are we doing anything positive?” Another staff member’s anxiety exacerbated to the point of panic: “I’ve had anxiety, but I’ve never had an attack until I got here, and it was like my heart was getting cold and I was like—am I going to pass out and die?”.

In response to these burdens, some Veterans who were anticipated to pose challenges were denied admission. There was concern other programs exploited CTRS, i.e., “dumping” their difficult cases. As one staff described: “We have Veterans with unique medical or mental health needs and there’s a gap in service where no one can come out and [provide direct clinical care]…I think access to a psychiatrist, boots on the ground who was willing to see Veterans and even do that street medicine approach…because we don’t have dedicated staff.” This view solidified the idea that providing clinical services within the encampment would not only improve access to care—as desired by Veterans—but would also improve staff wellness.

One Veteran told us CTRS Veterans and staff suffered low morale: “You can look at…their faces—our faces—and…you don’t see a lot of smiling…that’s one thing that could really use some help and improvement is building morale.” Some Veterans perceived staff burnout as a lack of empathy and respect. One Veteran shared: “You want to create an environment of comfort for those who serve…come from a position of empathy.”

## DISCUSSION

 A sense of stability and home were foundational to improving access to healthcare and housing services among unsheltered VEH. This vulnerable group needed a safe, secure, and consistent environment for living, sleeping, and storing personal belongings. VEHs also needed to trust providers and feel respected to develop the relationships necessary to access services. Though relationships built in CTRS allowed some VEHs to access care in brick-and-mortar settings, other VEHs desired on-site services.

As a result of a lack of consensus around the priorities of the intervention, staff varied in their conceptualization of CTRS and its goals with regard to enhancing VEHs’ access to healthcare. Tensions arose between VEHs who described CTRS as their first stable home in years—enabling increased healthcare access—and staff who viewed access to healthcare as outside the scope of an emergency shelter initiative. This lack of clarity, combined with the complex needs of CTRS residents, led to staff burnout, which may have impeded access to all services.

The role of stability in facilitating access to services is consistent with research indicating subsistence difficulties are barriers to health service utilization for unhoused adults.^32^ The importance of caring relationships for VEHs in this setting is also supported by studies showing unhoused adults report stigma, discrimination, and bias in traditional healthcare settings, including feelings of being shamed and stereotyped, particularly in the context of substance use.^20,21^ These experiences lead to avoiding care, not believing their concerns will be taken seriously, and a distrust of healthcare systems. The near universality of this point among CTRS residents emphasized the salience of trusting relationships as a prerequisite of accessing care.

Though CTRS leadership assumed residents would access care through the traditional on-campus services, Veterans and staff reported many residents were missing appointments or not accessing services. These barriers were due in part to past negative experiences, but also related to competing needs and disabilities making traveling to clinics and navigating virtual access challenging.

With an embedded research team, observations and feedback can be rapidly analyzed and disseminated for timely QI efforts. In the current study, presentations to CTRS personnel and VA leadership led to the development of opportunities for Veteran-led feedback to be more routinely shared. Monthly town hall meetings were organized to foster dialog between VA administration and staff and VEH to discuss ongoing challenges. Additionally, a weekly CTRS Veteran Engagement Committee was established where VEHs’ could voice concerns and recommendations.

The QI team is currently piloting an on-site “encampment medicine” team integrating primary care, psychiatry, substance use, and case management. This approach builds on evidence that Assertive Community Treatment—a model of assertive outreach and field-based services—improves psychiatric symptoms and reduces substance use among people experiencing homelessness.^33^ Our encampment medicine pilot also builds on street medicine, a model that can increase access to healthcare; reduce substance use, ED visits, and hospitalizations; and cultivate trust with historically marginalized patients.^34^ In addition, the QI team increased Whole Health programming (see Table 1) to foster well-being.^35^

The encampment medicine team not only provides clinical services but may respond to staff burnout by alleviating the burden of mental and physical health crises. Key dimensions of burnout, including exhaustion, cynicism, and reduced personal efficacy, were rampant in staff interviews.^36^ These data align with research showing frontline homeless service workers experience high levels of burnout^37^ and mental health challenges.^38,39^ Targeted interventions to address burnout among homeless program staff may enhance staff wellness and improve VEHs’ access to care.

This project has limitations. CTRS is a unique, urban setting and findings may not extrapolate to less-resourced settings (e.g., outside the VA); suburban or rural communities; or healthcare systems without integrated social services. Furthermore, our qualitative data were not integrated with administrative data or survey findings capturing CTRS residents’ use of healthcare services. We did, however, combine extensive participant observations with interviews and engaged in purposive sampling paralleling the characteristics of CTRS’ overall population.

## Conclusions

As encampments continue to be a feature of urban landscapes, attention is needed to shape them into settings of patient-centered wrap-around services.^40^ CTRS highlights the importance of establishing a clear purpose for a novel housing program in order increase access to services for unsheltered individuals. A supported encampment can serve as a path for some unsheltered individuals to exit homelessness but is not meant to replace permanent supportive housing. Rather, supported encampments can provide a safe place to stay while facilitating and expediting access to healthcare and housing. Further research on housing outcomes and healthcare utilization would enhance this article’s findings. Recently, CTRS transitioned to a “pallet shelter” community on VA grounds, i.e., private lockable cabins with beds, electricity, heat, and air-conditioning. Lessons learned from CTRS’ iteration as a sanctioned encampment can guide service development in these pallet shelters and inform service provision in similar settings.
